# PICCs versus PORTs, an Australian perspective. Comment on *BJA Open* 2025; 13: 100377

**DOI:** 10.1016/j.bjao.2025.100424

**Published:** 2025-06-18

**Authors:** Stuart Walker, Louise Nott

**Affiliations:** Royal Hobart Hospital, Hobart, Australia

Editor—We thank the authors of this *post hoc* analysis and their contribution to the literature on the advantages and disadvantages of peripherally inserted central catheters (PICCs) and implanted port catheters (PORTs) in the treatment of breast cancer patients.^1^ As the study was conducted in Sweden, we would like to provide an Australian perspective on this important clinical decision.

Firstly, all PICCs in the study were inserted by specialist nurses. This is consistent with practice in Australian public hospitals, where specialist nurses typically perform insertions, with some involvement from anaesthetists and interventional radiologists. However, the situation in private hospitals in Australia is more challenging. There are financial disincentives to PICC insertion in the private sector which might be resolved with evidence from further studies that include patient-reported outcome measures (PROMS) and cost–benefit analyses. Private patients face significant out-of-pocket costs over time to cover nursing care, consumables, and associated services required for regular line maintenance. Beyond the financial burden, the inconvenience and ‘time toxicity’ for patients, many of whom are managing life-threatening or terminal illnesses, are substantial, as they must travel and wait for weekly PICC care. While Utas and colleagues^1^ highlight the inconvenience of PICCs, particularly focusing on showering, additional practical limitations deserve emphasis. These include restrictions on swimming, bathing, and certain sports, and the risk of accidental dislodgement, particularly for patients with young children. These factors collectively add to the burden of PICC use in oncology patients.

We also noted that 87% of the PORTs in the study were inserted by anaesthetists. This contrasts with typical Australian practice, where most insertions are performed by surgeons from various specialities or by interventional radiologists. The procedures in the study were performed efficiently, with a reported duration of 21–39 min, and predominantly under local anaesthetic (83%), with only one case requiring a general anaesthetic. Interestingly, the authors observed that the proportion of patients reporting the implantation as painful was twice as high for PORTs compared with PICCs. We wonder whether this finding might, at least in part, reflect insufficient time allowed for the local anaesthetic to take full effect before the procedure commenced. Achieving adequate anaesthesia for tunnelling the catheter can also present a technical challenge, potentially contributing to patient discomfort. Furthermore, when study participants were questioned 3 months after insertion, significantly more patients in the PORT group also reported a numerical rating scale (NRS) pain score ≥4 during either needle insertion (for PORT) or dressing change (for PICC) (*P*<0.001). Furthermore, the authors reported that PORTs caused more discomfort than PICCs 1 month after insertion (*P*=0.022), although this difference was no longer evident at 3 months. These findings are not unexpected, given that PORTs, despite being implanted, require repeated needle access for use. In clinical practice, we routinely apply topical anaesthetic agents such as EMLA® cream for patients with needle sensitivity to minimise discomfort during needle access. It is not clear whether this strategy was used in this study.

In this study, the majority of patients had early-stage, curative intent breast cancer. Most patients in this group require access for 5–6 months, occasionally up to 12 months, unlike patients with advanced disease who may require access for many years. This may be a further consideration for either PICC or PORT in patients with breast cancer.

The patient satisfaction questionnaires at 6 and 12 months had very low response rates, preventing meaningful analyses. This is particularly important as this young cohort of women (mean age 61 yr in the PICC group and 62 yr in the PORT group) may still be working, exercising, and caring for others, so that quality-of-life differences may not have been captured.

Finally, no cost-effectiveness analysis was available. Such analysis would be very useful in the current climate of cost-effectiveness.

In conclusion, while both PICCs and PORTs offer distinct advantages in managing central venous access for breast cancer patients, the decision between the two must consider not only clinical factors, but also the broader context of patient quality of life, treatment duration, and financial implications. We believe that future studies should incorporate a more comprehensive assessment of patient-reported outcomes, including long-term quality of life, and a robust cost-effectiveness analysis to better inform clinical decision-making. Ultimately, shared decision making factoring in patient preferences, clinical circumstances, and the economic landscape, will ensure the most effective and sustainable care for this diverse patient population.

## Declarations of interest

The authors declare that they have no conflicts of interest.
